# Simple binary segmentation frameworks for identifying variation in DNA copy number

**DOI:** 10.1186/1471-2105-13-277

**Published:** 2012-10-30

**Authors:** Tae Young Yang

**Affiliations:** 1 Department of Mathematics, Myongji University, Yongin, Kyonggi, 449-728, Korea

**Keywords:** Bayesian information criterion, Circular binary segmentation, Consensus molecular signature, Overall molecular signature, Variation in DNA copy number

## Abstract

**Background:**

Variation in DNA copy number, due to gains and losses of chromosome segments, is common. A first step for analyzing DNA copy number data is to identify amplified or deleted regions in individuals. To locate such regions, we propose a circular binary segmentation procedure, which is based on a sequence of nested hypothesis tests, each using the Bayesian information criterion.

**Results:**

Our procedure is convenient for analyzing DNA copy number in two general situations: (1) when using data from multiple sources and (2) when using cohort analysis of multiple patients suffering from the same type of cancer. In the first case, data from multiple sources such as different platforms, labs, or preprocessing methods are used to study variation in copy number in the same individual. Combining these sources provides a higher resolution, which leads to a more detailed genome-wide survey of the individual. In this case, we provide a simple statistical framework to derive a consensus molecular signature. In the framework, the multiple sequences from various sources are integrated into a single sequence, and then the proposed segmentation procedure is applied to this sequence to detect aberrant regions. In the second case, cohort analysis of multiple patients is carried out to derive overall molecular signatures for the cohort. For this case, we provide another simple statistical framework in which data across multiple profiles is standardized before segmentation. The proposed segmentation procedure is then applied to the standardized profiles one at a time to detect aberrant regions. Any such regions that are common across two or more profiles are probably real and may play important roles in the cancer pathogenesis process.

**Conclusions:**

The main advantages of the proposed procedure are flexibility and simplicity.

## Background

Copy number variations (CNVs) in DNA, due to gains and losses of chromosome segments, is common among healthy individuals and an important feature of tumor genomes. In healthy individuals, CNVs (most of which are inherited) are usually short and spaced far apart, whereas in tumor subjects, they can be quite long, sometimes spanning entire chromosomes. Because genomic instability can trigger the overexpression or activation of oncogenes and the silencing of tumor suppressors, mapping regions of common genomic aberrations have been used to discover cancer-related genes. Understanding genome aberrations is important for a basic understanding of cancer, as well as for diagnosis and clinical practice [1,2]. CNVs from cancer tissues, referred to as copy number aberrations (CNAs), are acquired somatic aberrations most often observed only in cancer tissues. There is significant interest in locating CNVs in normal individuals and CNAs in tumor subjects [3].

Various microarray technologies, including array comparative genomic hybridization (aCGH), Affymetrix single-nucleotide polymorphism (SNP) genotyping arrays, Illumina Infinium arrays, and other SNP arrays, are used to investigate the roles of CNVs/CNAs. Here we describe aCGH in detail [4,5]. In this technique, DNA from a test sample and a normal reference sample are labeled differentially, using different fluorophores, and hybridized to several thousand spots on microarray chips. The spots are derived from most of the known genes and non-coding regions of the genome, printed on a glass slide. The recorded value for each probe in a given sample is usually the log_2_ ratio of the copy number measurement at the probe to its reference value, often computed from a set of normal population controls. The log_2_ratio of the normal state, in which the copy number of the target agrees with that of the control, should have a mean equal to zero. A contiguous stretch of measurements that are on average higher or lower than zero suggests a gain or loss in copy number.

The analysis of DNA copy number data consists of identifying amplified or deleted regions in each individual. There can be multiple CNVs/CNAs in a chromosome from a single sample. The binary segmentation procedure proposed by Vostrikova [6] has been widely used for locating multiple change-points. In each stage of this procedure, a single-change-point model is compared to a constant model with no change-points. Thus, the procedure is easily implemented and circumvents the computational complexity normally faced in problems with a variable number of change-points. A potential problem with the binary segmentation procedure is that it cannot detect a small segment buried in the middle of a large segment. Olshen et al. [7] modified the binary segmentation procedure to compare a model with a pair of change-points to a constant model with no change-points in each stage. This modified procedure is called circular binary segmentation, which is particularly useful for detecting short regions of a chromosome [7]. This approach recursively splits chromosomes into segments based on a statistic similar to the Student statistic, whose p-value is estimated by a time-consuming permutation process. To locate multiple CNVs/CNAs, we propose using circular binary segmentation based on a sequence of nested hypothesis tests, each using the Bayesian information criterion (BIC) [8]. Note that our version is based on the existing circular binary segmentation strategy, but the proposed BIC is computationally simple, and is different from previous methods. Various authors [9-11] have suggested a BIC criterion for determining the number of change-points.

In Methods Section, we describe the derivation of the proposed procedure and present a numerical example and simulation study. The proposed procedure can be flexibly adapted to analyze multiple DNA copy number data sets to discover both consensus and overall molecular signatures. In Results Section, these two general situations are separately discussed in “Integration of multiple platforms” and “Cohort analysis of multiple individuals”.

## Methods

Let *x*_*i*_ denote the log_2_ratio of the copy number measurement at the i-th probe of an individual. The vector ***X ***= (*x*_1_,…,*x*_*m*_) is then a DNA copy number data set for one chromosome of the individual, arranged according to genomic order along the chromosome.

For a given threshold *τ*^+ ^> 0, we construct a Bernoulli data set ***A ***= (*a*_1_,…,*a*_*m*_) for gain events such that

(1) $$a_{i}=1\;\text{if}\;x_{i}>\tau^{+}\;\text{and}\;a_{i}=0\;\text{otherwise}.$$

In a hypothetical situation for aCGH, Pollack et al. [12] specified log_2_0.8 ≤ log_2_ ratio <log_2_1.2 (-0.32 to 0.26) for the normal state, log_2_1.2 ≤ log_2_ ratio < log_2_2.0 (0.26 to 1) for low amplification, log_2_2.0 ≤ log_2_ ratio < log_2_3.0 (1 to 1.58) for medium amplification, and log_2_ratio > log_2_3.0 (=1.58) for high amplification. To locate low, medium, and high amplification, we would use *τ*^+^=0.32, 1, and 1.58, respectively. If there are gain events in the target chromosome of the individual, we expect to see many consecutive 1s in ***A***.

For a given threshold *τ*^− ^< 0, we create ***D ***= (*d*_1_,…,*d*_*m*_) such that

(2) $$d_{i}=1\;\text{if}\;x_{i}<\tau^{-}\;\text{and}\;d_{i}=0\;\text{otherwise}.$$

Pollack et al. [12] also specified log_2_ratio < log_2_0.8 (=-0.32) for loss. We would use *τ*^− ^= −0.32. If there are loss events in the target chromosome, we expect to see many consecutive 1s in ***D***.

The search for gain events is performed separately from that for loss events. To detect gain (loss) regions for an individual, we apply the following procedure to ***A***(***D***).

### Circular binary segmentation procedure

We assume that the success rate for a Bernoulli data set ***A*** at probe location *i* changes according to

$$p(i)=\overset{K+1}{\underset{k=1}{\sum }}\delta (i\in [c_{k-1}+1,c_{k}])\;p_{k}$$

where *δ*(*E*) is the indicator function for event *E* and 0 =* c*_0 _<* c*_1 _< ⋯ <* c*_*K *_<* c*_*K* + 1 _=* m *are the unknown integer-valued change-points with associated success rates *p*_1_,…,*p*_*K* + 1_. The goal of the change-point problem is to identify the number of change-points *K*, the change-points *c*_1_,…,*c*_*K*_, and the associated success rates *p*_1_,…,*p*_*K* + 1_.

We let M_0_ denote the constant model with no change-points (i.e. *θ*_0 _=* p*_1 _= ⋯ =* p*_*m*_). In M_0_, the likelihood is

(3) $$L_{0}(\theta_{0}|A)=\theta_{0}^{\sum_{i=1}^{m}a_{i}}{\left(1-\theta_{0}\right)}^{\sum_{i=1}^{m}(1-a_{i})}.$$

Using the circular binary segmentation procedure, we reduce the complexity of the problem by assuming that the segment forms a circle. We test the hypothesis that the arc from *c*_1_ + 1 to *c*_2_ and its complement have different success rates. Let M_1_ denote the change-point model given by a pair of *c*_1_and *c*_2_. This implies that *θ*_1 _=* p*_1 _= ⋯ = $\left(p_{c_{1}}=p_{c_{2}+1}=\cdots =p_{m}\neq p_{c_{1}+1}=\cdots =p_{c_{2}}=\theta_{2}\right)$, where 1 ≤* c*_1 _<* c*_2 _≤* m*. In M_1_, the likelihood is

(4) $$\begin{array}{l} L_{1}(c_{1},c_{2},\theta_{1},\theta_{2}|A)=\theta_{1}^{\overset{c_{1}}{\underset{i=1}{\sum }}a_{i}+\overset{m}{\underset{i=c_{2}+1}{\sum }}a_{i}}\\ \;\;\;\;\;\times {\left(1-\theta_{1}\right)}^{\overset{c_{1}}{\underset{i=1}{\sum }}(1-a_{i})+\overset{m}{\underset{i=c_{2}+1}{\sum }}(1-a_{i})}\\ \;\;\;\;\;\times \theta_{2}^{\overset{c_{2}}{\underset{i=c_{1}+1}{\sum }}a_{i}}{\left(1-\theta_{2}\right)}^{\overset{c_{2}}{\underset{i=c_{1}+1}{\sum }}(1-a_{i})}.\; \end{array}$$

Let us consider the constant model *M*_0_. The likelihood function (3) is maximized by $\left({\hat{\theta }}_{0}=\overset{m}{\underset{j=1}{\sum }}a_{j}/m\right)$, giving $\left(L_{0}({\hat{\theta }}_{0}|A)\right)$. For *M*_1_, the likelihood (4) is maximized along 1 ≤* c*_1 _<* c*_2 _≤* m *via

$$\begin{array}{ll} \left({\hat{\theta }}_{1}(c_{1},c_{2}),{\hat{\theta }}_{2}(c_{1},c_{2})\right)=\left(\frac{\overset{c_{1}}{\underset{i=1}{\sum }}a_{i}+\overset{m}{\underset{i=c_{2}+1}{\sum }}a_{i}}{m-c_{2}+c_{1}},\right)\\ \;\;\;\;\;\left(\frac{\overset{c_{2}}{\underset{i=c_{1}+1}{\sum }}a_{i}}{c_{2}-c_{1}}\right).\; & \; \end{array}$$

The fully maximized likelihood in the segmentation model $\left(L_{1}({ĉ}_{1},{ĉ}_{2},{\hat{\theta }}_{1}({ĉ}_{1},{ĉ}_{2}),{\hat{\theta }}_{2}({ĉ}_{1},{\hat{c}}_{2})|A)\right)$ is then obtained by maximizing $\left(L_{1}(c_{1},c_{2},{\hat{\theta }}_{1}(c_{1},c_{2}),{\hat{\theta }}_{2}(c_{1},c_{2})|A)\right)$ over the finite set 1 ≤* c*_1 _<* c*_2 _≤* m*.

We choose between M_0 _and M_1_ in accordance with the BIC. We define

(5) $$\begin{array}{l} {\text{BIC}}_{10}=\text{log}L_{1}({ĉ}_{1},{ĉ}_{2},{\hat{\theta }}_{1}({ĉ}_{1},{ĉ}_{2}),{\hat{\theta }}_{2}({ĉ}_{1},{ĉ}_{2})|A)\\ \;\;\;-\text{log}L_{0}({\hat{\theta }}_{0}|A)-\frac{1}{2}(q_{1}-q_{0})\text{log}\;m \end{array}$$

where the last term in (5) is a penalty function that adjusts for the difference in dimensionality between the two models. In this application, *q*_1 _= 4 and *q*_0 _= 1. If BIC_10_ is negative, the decision is to accept M_0_. If BIC_10 _is positive, we reject the constant model and estimate the first segment given by the pair of $\left({ĉ}_{1}\right)$ and $\left({ĉ}_{2}\right)$.

To test *M*_0_ versus *M*_1_, the procedure begins by setting *c*_1 _= 1 and *c*_2 _=* m*. Let $\left({\text{BIC}}_{10}^{\text{obs}}\right)$ be the observed BIC_10_, and $\left(\left[{ĉ}_{1}+1,{ĉ}_{2}\right]\right)$ be the corresponding interval. If $\left({\text{BIC}}_{10}^{\text{obs}}<0\right)$, we choose *M*_0_, estimate the constant success rate to be $\left(\hat{p}(i)={\hat{p}}_{1}\right)$ for *i *∈[1,*m*] with $\left({\hat{p}}_{1}=\overset{m}{\underset{j=1}{\sum }}a_{j}/m\right)$, and stop. If $\left({\text{BIC}}_{10}^{\text{obs}}>0\right)$, $\left(\left[1,{ĉ}_{1}\right]\right)$, $\left(\left[{ĉ}_{1}+1,{ĉ}_{2}\right]\right)$, and $\left(\left[{ĉ}_{2}+1,m\right]\right)$ are recursively scanned using the same procedure. The recursion stops when none of the subregions contains its corresponding $\left({\text{BIC}}_{10}^{\text{obs}}>0\right)$.

### Application to aCGH data

**Figure 1:**
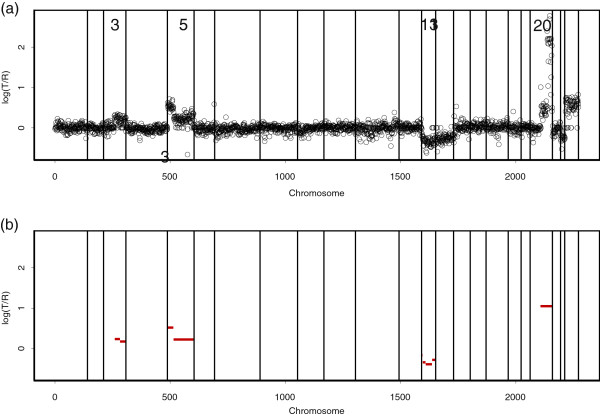
**The breast cancer S1514. (a)** The points are normalized log_2_ratios. The BACs are ordered by position in the genome, beginning at 1p and ending at Xq. The inserts are chromosome numbers. The borders between chromosomes are represented by vertical bars. **(b)** Our analysis of S1514. The normal state, where the copy number in the target agrees with that in the control, should have mean 0. A contiguous range of measurements whose average is higher or lower than 0 suggests a respective gain or loss in copy number. To identify gains and losses, we used *τ*^+ ^= 0.3 and *τ*^− ^= −0.3, respectively. We found single-copy duplication from the center to the end of chromosome 3. We identified double-copy duplication at the beginning of chromosome 5 and single-copy duplication in the remaining region of chromosome 5. We also identified very high-level amplification from the center to the end of chromosome 20. We found low-level losses on chromosome 13. The red lines indicate the mean values among the probes in segments detected by our method.

Snijders et al. [5] used aCGH to detect low-level DNA copy number gains and losses, as well as high-level amplifications for breast cancer specimen S1514. Their array contained 2276 probes for the mapped bacterial artificial chromosomes (BACs), which are large segments of DNA, typically 100 to 200 kilo-bases. Figure 1(a) shows a plot of the normalized log_2_ ratios of S1514. Low-level gains and losses, as well as high-level amplifications were found in S1514.

In Figure 1(b), we respectively use *τ*^+ ^= 0.3 in Equation (1) and *τ*^− ^= −0.3 in Equation (2) to identify gains and losses. Our procedure was executed to detect aberrated regions for each of the 23 chromosomes. The red lines indicate the mean values among clones in segments obtained by our procedure. We found gains on chromosomes 3 and 5, loss on chromosome 13, and high-level amplification on chromosome 20.

**Figure 2:**
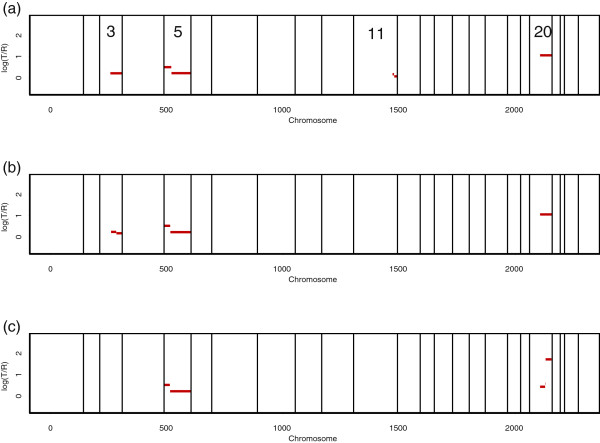
**Our analysis of the breast cancer S1514.** The borders between chromosomes are represented by vertical bars. The red lines indicate the mean values among the probes in segments obtained by the proposed procedure. As we increase the value of *τ*^+^, higher-level gains are readily identifiable. **(a)** For *τ*^+ ^= 0.2, we identified single-copy duplication at the end of chromosomes 3 and 11. We identified double-copy duplication at the beginning of chromosome 5 and single-copy duplication in the remaining region of chromosome 5. We also identified very high-level amplification from the center to the end of chromosome 20. **(b)** For *τ*^+ ^= 0.3, we found single-copy duplication from the center to the end of chromosome 3. We identified double-copy duplication at the beginning of chromosome 5 and single-copy duplication in the remaining region of chromosome 5. We also identified very high-level amplification from the center to the end of chromosome 20. We did not identify alternations on chromosome 11, due to low-level amplified signals. **(c)** For *τ*^+ ^= 0.5, we identified double-copy duplication in the center of chromosome 20. At the end of chromosome 20, we identified very high-level gain (more than triple-copy). We identified double-copy duplication at the beginning of chromosome 5 and single-copy duplication in the remaining region of chromosome 5. We did not identify alternations on chromosomes 3 and 11, due to low-level amplified signals.

**Figure 3:**
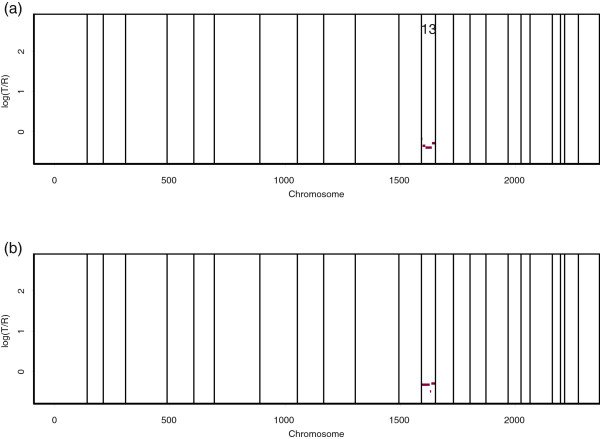
**Our analysis of the breast cancer S1514.** The borders between chromosomes are represented by vertical bars. The red lines indicate the mean values among the probes in segments obtained by the proposed procedure. **(a)** For *τ*^− ^= −0.3, we identified single-copy loss at chromosome 13. **(b)** For *τ*^− ^= −0.4, we identified single-copy loss at chromosome 13.

As we increase *τ*^ + ^, higher-level gains are readily identifiable, as shown in Figure 2. As we decrease *τ*^−^, lower-level losses are readily identifiable, as shown in Figure 3. From Figure 2 and Figure 3, amplified and deleted regions of an individual are clearly separated, because these regions would trigger the activation of oncogenes and the silencing of tumor suppressors, respectively.

### Simulation study

We evaluated the performance of our algorithm. The data to be segmented were generated from the model *x*_*i*_∼*N*(*μ*_*i*_,1),1 ≤* i *≤* m*, where *m* is the number of probes and *μ *denotes the mean. Let *μ*_*i *_=* c *when *l *<* i *≤* l* + *k*, and *μ*_*i *_= 0 otherwise. The mean parameter *c* was set equal to 1, 2, or 3. The value *c *= 1 represents low-level amplification. The values *c *= 2 and *c *= 3 represent moderate and high-level amplification, respectively. We simulated 1000 data sets from 500 probes using this simulation setup.

We randomly selected *k* from (3,…,30), and *l* from (1,2,…,*m*−*k*). The values of *l* and *k* control the location of the change and the width of the changed segment, respectively. Note that the width of the changed segment is at least 3 probes. Each data set had one elevated region ranging from 3-30 probes, and the elevation varied according to *c*.

The power is the proportion of data sets in which the estimated change-points equal the true change-points. Table 1 lists the power for various *c* and *τ*^ + ^. The power was lower for *c *= 1 because *c *= 1 represents low-level amplification. However, it increased as *c* increased.

**Table 1:** **Power for various *τ***^+^** and *c***

***c***	***τ***^**+**^							
	**0.5**	**1.0**	**1.5**	**2.0**	**2.5**	**3.0**		
1	0.67	0.68	0.67	0.64	0.40	0.27		
2	0.93	0.95	0.98	0.98	0.91	0.87		
3	0.96	1	1	1	1	1		

The numbers represent proportions of 1000 predetermined elevated regions identified by our method.

When *τ*^+ ^≤ 2.0, we identified low- and higher-level amplification, and thus the power was high. In contrast, when *τ*^+ ^≥ 2.5, we only observed higher-level amplification as *τ*^+ ^increased, and consequently the power was lower.

## Results

### Integration of multiple platforms

Several sources (platforms, analytical methods, and labs) were used to study the variation in copy number of the same individual. Their profiles may have different mean levels of copy number aberrations and different noise levels [13,14]. They may also have different numbers of loci and variable coverage in different parts of the genome. If data sets from several sources are analyzed individually, it is difficult to reach a consensus when they disagree on the identity of a CNV/CNA. Combining data sets may increase resolution, facilitating the discovery of genes and probes that are important in the individual. To derive a consensus molecular profile, we combine multiple sources into a single sequence, and then apply our procedure to this sequence.

The observed data constitute a two-dimensional array *x*_*ij*_ for *i *= 1,…,*m*_*j*_ and *j *= 1,…,*n*, where *x*_*ij*_ is the data point at the *i*-th probe and the *j*-th source, and *n* is the total number of sources. For the *j*-th source, *m*_*j*_ probes are ordered by chromosome location $\left(\left(t_{1j},\ldots ,t_{m_{j}j}\right)\right)$, which may have variable loci and coverage in parts of the chromosome.

For a given threshold $\left(\tau_{j}^{+}>0\right)$, an indicator variable *a*_*ij *_is defined to classify the DNA copy number level as increased or not; i.e.,

(6) $$a_{\text{ij}}=1\;\text{if}\;x_{\text{ij}}>\tau_{j}^{+}\;\text{and}\;a_{\text{ij}}=0\;\text{otherwise}.$$

We then construct a Bernoulli data set $\left(A_{j}=(a_{1j},\ldots ,a_{m_{j}j})\right)$ for each source *j*. Because different sources exhibit different degrees of attenuation of the true DNA copy number, we use a threshold $\left(\tau_{j}^{+}\right)$ for each source, rather than applying a common threshold to all sources. Note that we do not require pre-standardization of different sources. We keep these sequences ordered according to chromosome position, and integrate $\left(\left(t_{11},\ldots ,t_{m_{1}1}),\ldots ,(t_{1n},\ldots ,t_{m_{n}n}\right)\right)$ into a single sequence, which is the union of the chromosomic locations of probes from all profiles. Then ***A***_1_,…,***A***_*n*_ are integrated into ***A*** along the single sequence. ***A ***provides a consensus molecular profile and higher resolution for detecting CNAs. If there are amplification events in the target chromosome, we expect to see many consecutive 1s in ***A***. To identify amplification regions, we apply the proposed procedure to ***A***, as discussed in Methods Section.

The search for loss events is performed separately from that for gain events. For a given threshold $\left(\tau_{j}^{-}<0\right)$ and for each source *j*, *d*_*ij *_is defined to classify the DNA copy number level as decreased or not:

(7) $$d_{\text{ij}}=1\;\text{if}\;x_{\text{ij}}<\tau_{j}^{-}\;\text{and}\;d_{\text{ij}}=0\;\text{otherwise.}$$

We then construct a Bernoulli data set $\left(D_{j}=(d_{1j},\ldots ,d_{m_{j}j})\right)$ for *j *= 1,…,*n*, and ***D***_1_,…,***D***_*n*_ are integrated into ***D***along the integrated single sequence. To identify deletion regions for the individual, we apply the proposed procedure to ***D***.

#### Application to The Cancer Genome Atlas data

The Cancer Genome Atlas (TCGA) project ( http://tcga-data.nci.nih.gov/tcga) is a collaborative initiative for a better understanding of cancer, using existing large-scale complete-genome technologies [15]. One of the tumor types studied is glioblastoma multiforma (GBM), which is a brain tumor. The TCGA-02-0104 (vials 01A) sample is known to have a large number of copy number aberrations on chromosome 3 at different mean levels [13]. To provide an application to somatic CNAs, we analyze TCGA-02-0104 samples from two TCGA centers: the Memorial Sloan-Kettering Cancer Center and Harvard Medical School. Both centers adopted Agilent CGH 244 K arrays, which have 236000 loci, 12.7 kb average between loci, and 60-mer probes. The different TCGA centers have identified aberrant regions independently of one another. It has been suggested that more accurate, precise, and higher-resolution results could be obtained if copy number estimates from the different sites were combined.

**Figure 4:**
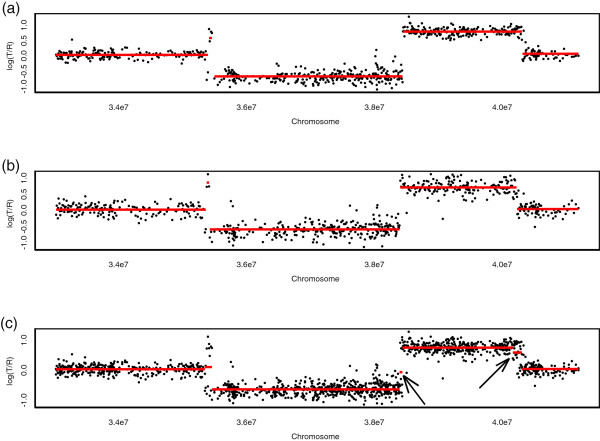
**Consensus estimate.** The points are normalized log_2_ratios in the 33-42-mb section on chromosome 3 of the TCGA-02-0104 sample from **(a)** the Memorial Sloan-Kettering Cancer Center (MSKCC) and **(b)** Harvard Medical School. The red lines indicate the mean values among the probes in segments obtained by the proposed circular binary segmentation procedure. Panel **(c)** shows a multi-platform consensus based on the combined data sets of Memorial Sloan-Kettering Cancer Center and Harvard Medical School. We found two more small segments, located in the 38.4-mb and 40.2-mb regions, which are indicated by the arrows. These were not identified in **(a)** and **(b)**.

The proposed procedures were separately applied to detect amplification or deletion in the 33-42-mb (start-end) region on chromosome 3. Figure 4(a) and 4(b) show the individual results of the Memorial Sloan-Kettering Cancer Center and Harvard Medical School, respectively. Here, we used $\left(\tau_{1}^{+}=\tau_{2}^{+}=0.5\right)$ in Equation (6) and $\left(\tau_{1}^{-}=\tau_{2}^{-}=-0.5\right)$ in Equation (7) because the two centers used the same Agilent platform. Figure 4(c) shows a consensus estimate along the integrated sequence. We found two short fluctuations, located in the 38.4-mb region and the 40.2-mb region, as indicated by the arrows in the figure. Note that these two segments were not identified by the single-source analyses presented in Figure 4(a) and 4(b).

**Figure 5:**
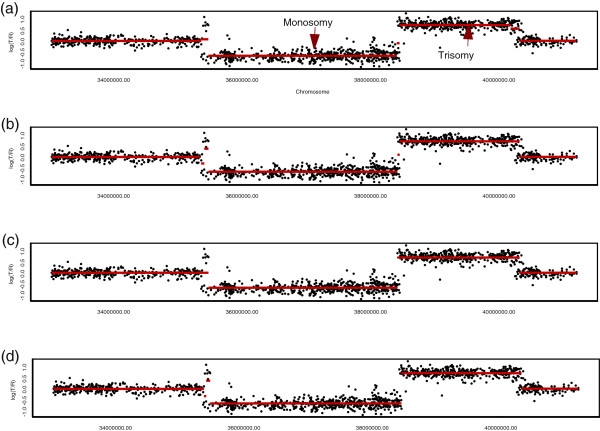
**Comparison of the proposed method, circular binary segmentation, CSGseq, and GLAD.** The points are based on the combined log_2_ratios from Memorial Sloan-Kettering Cancer Center and Harvard Medical School. The top panel shows our segments. The last three panels show segments from circular binary segmentation, CSGseq, and GLAD. The red lines indicate the mean values among the probes in segments.

In Figure 5, our results are compared with popular CNV segmentation algorithms including circular binary segmentation [7], CGH-seg [16], and GLAD [17]. Their segment results are obtained by a web-based tool, CGHweb [18]. All methods show that gain and loss regions are respectively 35-38 mb (3p22.2-3p22.3) and 38-40 mb (3p22.1-3p22.2). However, our method and circular binary segmentation [7] are sensitive to the detection of short segments in this example.

Circular binary segmentation [7] based on permutation took 95 seconds to detect the segmentation results of a total of 1358 probes, as shown in Figure 5. In contrast, the proposed procedure based on BIC took less than 15 seconds, where the computation was done on a 2.66 GHz Intel i5 core processor.

### Cohort analysis of multiple individuals

We turn next to the cohort problem of discovering overall molecular signatures. Each profile is obtained from a different individual with the same type of cancer, and is assayed on the same platform type. The observed data are a two-dimensional array *x*_*ij*_ for *i *= 1,…,*m*,*j *= 1,…,*n*, where *x*_*ij*_ is the data point at the *i*-th probe according to its genomic order along the chromosome, and the *j*-th individual profile. Note that *m* is the number of probes and *n* is the number of individuals. To derive overall molecular signatures, we provide a simple statistical framework, which standardizes data across multiple profiles before segmentation. Then, we analyze the standardized profiles one at a time to detect aberrant regions.

We standardize *x*_*ij*_. For each probe *i*, we let $\left(z_{\text{ij}}=\left(x_{\text{ij}}-\frac{\overset{n}{\underset{j=1}{\sum }}x_{\text{ij}}}{n}\right)/\sqrt{\frac{\overset{n}{\underset{j=1}{\sum }}{\left(x_{\text{ij}}-\overset{n}{\underset{j=1}{\sum }}x_{\text{ij}}/n\right)}^{2}}{n-1}}\right)$ for *j *= 1,…,*n*. Hence the *z*_*ij *_have a common mean equal to 0 and a common variance equal to 1. An indicator variable *a*_*ij*_ is defined to classify the DNA copy number level for the *i*-th probe and *j*-th individual as increased or not; i.e.,

(8) $$a_{\text{ij}}=1\;\text{if}\;z_{\text{ij}}>\gamma^{+}\;\text{and}\;a_{\text{ij}}=0\;\text{otherwise}.$$

For the following numerical example, we used *γ*^+ ^= 3. A segment with probes deviating by three standard deviations from the mean value of all samples is likely to indicate true gain. For large *γ*^+^, higher-level gains are readily identifiable. If there are gain events in the target chromosome of the *j*-th individual (*j *= 1,…,*n*), we expect to see many consecutive 1s in ***A***_*j *_= (*a*_1*j*_,…,*a*_*mj*_). To identify the amplification regions for the *j*-th individual, we apply the proposed procedure to ***A***_*j*_, as discussed in Methods Section. When common amplified regions occur for more than one individual, the aberrations are probably real and important for cancer pathogenesis processes.

The search for loss events is performed separately from that for gain events. *d*_*ij*_ is defined to classify the DNA copy number level for the *i*-th probe and the *j*-th individual as decreased or not; i.e.,

(9) $$d_{\text{ij}}=1\;\text{if}\;z_{\text{ij}}<\gamma^{-}\;\text{and}\;d_{\text{ij}}=0\;\text{otherwise}.$$

For our numerical example, we used *γ*^− ^= −3. If there are deletion events in the target chromosome of the *j*-th individual, we expect to see many consecutive 1s in ***D***_*j *_= (*d*_1*j*_,…,*d*_*mj*_). To identify the deletion regions of the *j*-th individual, we apply the proposed procedure to ***D***_*j*_.

Standardization across multiple samples provides a multi-sample summary for the overall molecular signatures. However, one drawback to this type of standardization is that it restricts inferences about increased and decreased DNA copy numbers relative to the mean of the samples under study. When most or all samples are either two-fold over-expressed or under-expressed relative to normal tissue (i.e., a majority of the samples have identical increases or decreases), it is impossible to properly identify these aberrations using the proposed standardization. These situations are very rare, and most aberrant intervals appear only in some significant subset of the samples. When pooling data across multiple individuals, not all samples are expected to carry the same aberrant regions.

#### Application to fibroblast cell lines

**Figure 6:**
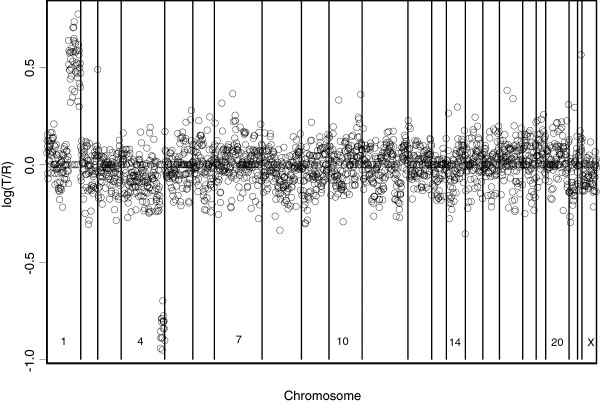
**The fibroblast cell line GM13300 has known alterations only on chromosomes 1 and 4.** The points are normalized log_2_ratios. The borders between chromosomes are represented by vertical bars.

**Figure 7:**
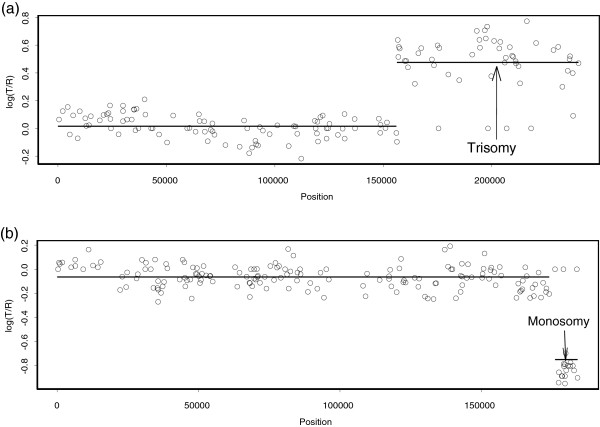
**Our analysis of GM13300.** The fibroblast cell line GM13300 has known alterations only on chromosomes 1 and 4. The points are normalized log_2_ratios, and the lines indicate the mean values among the points in segments obtained by our method. **(a)** CNVs of GM13300 on BAC clones from chromosome 1. **(b)** CNVs of GM13300 on BAC clones from chromosome 4.

**Figure 8:**
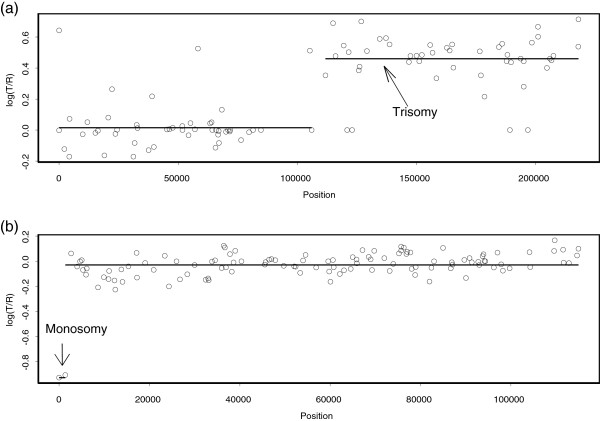
**Our analysis of GM03563.** The fibroblast cell line GM03563 has known alterations only on chromosomes 3 and 9. The points are normalized log_2_ratios, and the lines indicate the mean values among the points in segments obtained by our method. **(a)** CNVs of GM03563 on BAC clones from chromosome 3. **(b)** CNVs of GM03563 on BAC clones from chromosome 9. The first two clones with log_2_ratio≈ -1 indicate a single-copy deletion.

We applied our framework to the aCGH data presented by Snijders et al. [5]. The data were obtained from single-array experiments on 15 fibroblast cell lines. The data are available in Tables E to H at http://www.nature.com/ng/journal/v29/n3/suppinfo/ng754_S1.html. Each array contains 2276 mapped BACs spotted in triplicate. Because spectral karyotyping has shown that aberrations occur within a particular chromosome for each of GM01524, GM01535, GM01750, GM03134, GM03563, GM05296, GM07081, GM13031, and GM13330, we limited our analysis to these nine cell lines. The data from a typical cell line experiment, specifically from cell line GM13300, can be seen in Figure 6. The proposed procedure was employed to detect aberrated regions for each of the 23 chromosomes. We used *γ*^+ ^= 3 in Equation (8) and *γ*^− ^= −3 in Equation (9), respectively. GM13300, shown in Figure 6, has known aberrations only on chromosomes 1 and 4. The results shown in Figure 7 are consistent with those of Snijders et al. [5], in that our framework correctly identified aberrations only on chromosomes 1 and 4. Our procedure also correctly identified aberrations on chromosomes 3 and 9 of GM03563 (Figure 8).

Of the 15 aberrated regions listed in Table 2, which were found by spectral karyotyping, 13 were identified by our framework. The two unidentified regions were on chromosome 12 (GM01535) and chromosome 15 (GM07081). The aberrated region on GM01535 is represented by only a single probe, and single aberrated probes cannot be found. For GM07081, our result is consistent with that of Snijders et al. [5], in that no evidence of an aberration appears in the aCGH data. Therefore, our procedure found everything it should have found. For a particular cell line and chromosome, we define a false positive to be an aberration that is identified by our framework but is not detected by spectral karyotyping. Our procedure produced only one false positive, at chromosome 4 on GM01524, although we do not know that this is a real aberration that is undetectable by spectral karyotyping. Hence, our procedure was able to identify the aberrations with only a single false positive, whereas the circular binary segmentation method of Olshen et al. [7] produced at least nine false positives. Furthermore, the aberrations identified by our procedure perfectly matched the CNVs found via spectral karyotyping.

**Table 2:** Summarized results of applying the proposed framework to nine cell lines

**Cell line**	**Chromosome (exact location)**	**Aneuploidy type**	**Our method**
GM03563	3 (3q12-3qter)	Trisomy	O
	9 (9pter-9p24)	Monosomy	O
GM05296	10 (10q21-10q24)	Trisomy	O
	11 (11p12-11p13)	Monosomy	O
GM01750	9 (9pter-9p24)	Trisomy	O
	14 (14pter-14q21)	Trisomy	O
GM03134	8 (8q13-8q22)	Monosomy	O
GM13330	1 (1q25-1qter)	Trisomy	O
	4 (4q35-4qter)	Monosomy	O
GM01535	5 (5q33-5qter)	Trismoy	O
	12 (12q24-12qter)	Monosomy	X
GM07081	7 (7pter-7q11.2)	Trisomy	O
	15 (15pter-15q11.2)	Monosomy	X
GM13031	17 (17q21.3-17q23)	Monosomy	O
GM01524	6 (6q15-6q25)	Trisomy	O

Of the 15 altered regions found by spectral karyotyping, 13 were identified by our method. The symbol O indicates that our method correctly identified the known alterations found by spectral karyotyping, whereas the symbol × indicates that it did not identify the region. Two unidentified regions appear in chromosome 12 on GM01535 and chromosome 15 on GM07081. For GM01535, the region is represented by only one probe, and single altered probes were not found. For GM07081, our result is consistent with that of Snijders et al. [5], in that no evidence of an alteration was seen in the GM07081 data.

## Discussion

Our procedure is versatile in the sense that only higher- or lower-level gains/losses are readily identifiable. In particular, there are two interesting types of aberrated regions. The first of these is a spike, which is often a small region with extremely large or small log_2 _ratios. Only spikes are readily identifiable when large positive values of *τ*^+ ^ and large negative values of *τ*^− ^are used in Equations (1) and (2), respectively. The second type is a consistent gain or loss region, whose log_2_ratios may not deviate very much from 0, but tend to remain positive or negative over the greater region. Only lower-level gains are readily identifiable when we define a new Bernoulli data set ***A ***= (*a*_1_,…,*a*_*m*_) for a small positive value of *τ*^+ ^ and a positive value of *ε*, such that

$$a_{i}=1\;\text{if}\;\tau^{+}<x_{i}<\tau^{+}+\varepsilon \;\text{and}\;a_{i}=0\;\text{otherwise}.$$

Similarly, only lower-level losses are readily identifiable for a small negative value of *τ*^−^ when we define a new Bernoulli data set ***D ***= (*d*_1_,…,*d*_*m*_) such that

$$d_{i}=1\;\text{if}\;\tau^{-}-\varepsilon <x_{i}<\tau^{-}\;\text{and}\;d_{i}=0\;\text{otherwise}.$$

**Figure 9:**
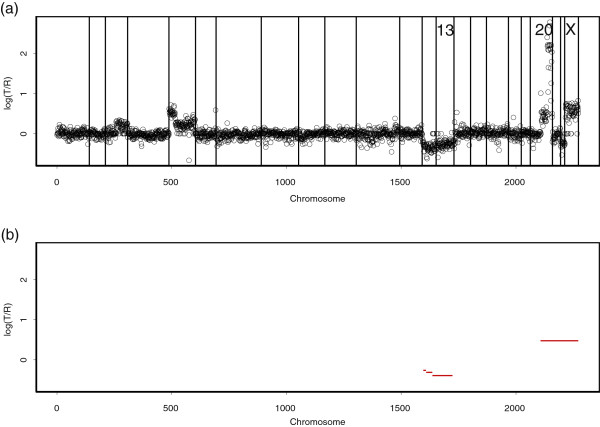
**Genome-wide analysis of the breast cancer S1514. (a)** The points are normalized log_2 _ratios. The BACs are ordered by position in the genome, beginning at 1p and ending at Xq. The inserts are chromosome numbers. The borders between chromosomes are represented by vertical bars. **(b)** Genome-wide search of S1514. To identify gains and losses, we used *τ*^+ ^= 0.3 and *τ*^− ^= −0.3, respectively. We found single-copy gain from the center of chromosome 20 to the end of 23 (chromosome X), and single-copy loss from the beginning of chromosome 13 to the end of chromosome 14. The red lines indicate the mean values among the probes in segments detected by our method.

We pointed out that our procedure lacks the ability to detect CNAs when a whole chromosome is duplicated or deleted. For example, in Figure 1, the elevated X chromosome ratios of S1514 reflect the male-female difference in the X chromosome copy numbers shown. These elevations are known to be constant for single-copy gains on a complete X chromosome. Because there were no fluctuations on the elevated, complete X chromosome, our procedure could not detect the aberrations when based on a chromosome-wide search. To detect aberrations spanning complete chromosomes, our procedure should be based on a genome-wide search, which uses all 23 chromosomes together. Figure 9 shows the genome-wide search, which properly identified single-copy duplication in the entire X chromosome.

## Conclusions

To locate the aberrated regions in an individual, we propose a circular binary segmentation procedure based on BIC, which is nonparametric in the sense that it does not rely on any assumptions regarding independence or underlying distributions. The procedure does not require data to be transformed with missing values imputed or with extreme outliers truncated. At each stage of the procedure, we need only to compare a model with a pair of change-points to a constant model with no change-points. Thus the procedure is easy to implement, and circumvents the computational complexity we would normally face in problems with a variable number of change-points. The procedure can be flexibly adapted to analyze multiple DNA copy number data sets, to discover consensus molecular signatures or overall molecular signatures. Moreover, we provide two simple statistical frameworks appropriate for detecting these signatures.

## Competing interests

The author declares that he have no competing interests.
